# Prenatal exposure to adverse life events and autism and autistic‐like traits in children in the Norwegian Mother, Father and Child Cohort Study (MoBa)

**DOI:** 10.1002/jcv2.70002

**Published:** 2025-03-22

**Authors:** Aleksandra Kanina, Arvid Sjölander, Miriam I. Martini, Agnieszka Butwicka, Henrik Larsson, Amanda M. Hughes, Márta K. Radó, Mark J. Taylor, Alexandra Havdahl, Helga Ask, Mina A. Rosenqvist

**Affiliations:** ^1^ Department of Medical Epidemiology and Biostatistics Karolinska Institutet Stockholm Sweden; ^2^ Division of Mental Health Services Akershus University Hospital Lørenskog Norway; ^3^ Institute of Clinical Medicine University of Oslo Oslo Norway; ^4^ Department of Biostatistics and Translational Medicine Medical University of Lodz Lodz Poland; ^5^ School of Medical Sciences Örebro University Örebro Sweden; ^6^ MRC Integrative Epidemiology Unit University of Bristol Bristol UK; ^7^ PsychGen Center for Genetic Epidemiology and Mental Health Norwegian Institute of Public Health Oslo Norway; ^8^ Department of Psychology PROMENTA Research Center University of Oslo Oslo Norway; ^9^ PaGE Research Group Lovisenberg Diaconal Hospital Oslo Norway

**Keywords:** autism, delayed effects, MBRN, MoBa, prenatal exposure, sibling‐comparison design

## Abstract

**Background:**

Mothers' experience of adverse life events (ALEs, e.g., divorce, bereavement, injury) during pregnancy has been linked with neurodevelopmental conditions like autism, and related traits like social communication difficulties and repetitive behavior in children. However, both the cumulative association and the underlying mechanism are unclear, and these associations might be confounded by unmeasured genetic or other early environmental factors shared within families.

**Method:**

This longitudinal population‐based cohort study included 114,247 children, born in Norway between 1999 and 2009, who participated in the Norwegian Mother, Father and Child Cohort Study. During week 30 of pregnancy, mothers of 51,940 children (of whom 12,597 were siblings) reported whether they had experienced ALEs. We estimated associations between mothers’ cumulative exposure to and perception of ALE and their children’s clinical diagnosis of autism, and maternal reports on their children's autistic traits at ages 3 and 8 years through the Social Communication Questionnaire (SCQ). Sibling comparisons were conducted to account for unmeasured familial confounding.

**Results:**

Each additional prenatal ALE was associated with increased adjusted hazard ratios [HR: 1.23, 95% CI: 1.16–1.30] of autism diagnosis, compared to unexposed children. Adjusting for unmeasured familial confounding in sibling comparisons, the association attenuated: HR = 0.53, 95% CI [0.31–0.90]. ALEs perceived as more painful were associated with a 12% elevated likelihood of autism diagnosis [95% CI: 7%–16%], but this association attenuated after sibling comparisons. SCQ scores in children exposed to cumulative prenatal ALE compared to unexposed children were higher at age 3 (β‐coefficient: 0.24 (95%CI [0.21–0.27])), but only slightly at age 8 (β‐coefficient: 0.07 [95% CI: 0.04–0.10]) with differences nullified in the sibling comparison analysis.

**Conclusion:**

The association between maternal prenatal exposure to cumulative ALEs and diagnosis of autism and autism‐associated traits is likely due to unmeasured familial confounding rather than a direct causal relationship.

**Key Points**

- Mothers’ experience of adverse life events (ALEs) during pregnancy has been linked with autism and autistic traits in children, but the effect of unmeasured genetic and environmental confounding is unclear.
- Based on a large longitudinal cohort study, we used sibling comparisons to account for unmeasured familial confounding in the association between ALEs during pregnancy and autism and autistic traits in their children.
- We found that cumulative exposure to ALEs during pregnancy was associated with autism and autistic traits in children, but the association attenuated after adjustment for familial confounding.
- This indicates that the association between ALEs during pregnancy and autism and autistic‐like traits in children is likely due to unmeasured familial confounding rather than a direct causal relationship.

## INTRODUCTION

Autism is a common neurodevelopmental condition, characterized by restricted and repetitive behaviors and difficulties with social interactions and communication. The global prevalence of diagnosed autism is estimated to be around 2% and has increased in recent years (Kim et al., 2011). Autism typically first manifests during early childhood. However, challenges faced by autistic individuals often remain unnoticed, resulting in a diagnosis being received during the later stages of adolescence or adulthood or remaining undiagnosed (Shattuck et al., 2009). Using continuous measures of autism‐associated traits could be advantageous to capture children who show autistic‐like traits and might experience autism‐related difficulties in everyday life but have not received a clinical diagnosis (Constantino & Todd, 2003). The complex nature of autism is universally acknowledged. While autism is highly heritable (60%–90%) based on twin studies (Tick et al., 2016), environmental factors have also been suggested to play a role in the emergence and clinical course of autism. These include factors associated with the mother's environment during pregnancy, low birth weight, and congenital malformations (Carlsson et al., 2021).

Research on associations between adverse life events (ALEs, also called stressful life events, or negative life events) reported by mothers during pregnancy and autism and autistic traits in their children is scarce and mainly focuses on separate events. For example, a large Swedish study (Class et al., 2014) showed that bereavement stress during pregnancy increased the likelihood of receiving an autism diagnosis. A small Canadian study (*N* = 33) showed that maternal experiences of objective hardship, such as threat, loss or change of environment, subjective distress and cognitive appraisal during pregnancy were associated with increased autism‐like traits in children (Li et al., 2023). However, the explanations for the observational association between prenatal ALE and autism are poorly understood.

While individual ALEs are associated with autism, it is not clear whether experiencing more than one ALE (cumulative exposure) during pregnancy would increase the association in a dose‐response pattern. The cumulative risks model proposes that developmental outcomes are influenced by the accumulation of predisposing factors, regardless of which specific predisposing factors are present (Berg et al., 2016). This means that with increasing number of predisposing factors, the likelihood of clinical outcomes increases (Rutter, 1979). The perception of the same event might differ between individuals. For example, a conflict at work could be perceived as very stressful by one individual and less stressful by another. Considering the subjective experience of an ALE can help take into account the difference between various ALEs: arguing with work colleagues might not be perceived as stressful as the death of a close relative. Studies exploring prenatal ALEs and autism have not accounted for the perceived experiences. However, the importance of accounting for the perception of ALEs was illustrated by a small cohort study reporting that subjective maternal distress during pregnancy explained 15% of the variance in children's pragmatic language impairment at the age of 19 (Li et al., 2023). Previous studies have reported an association between cumulative ALE exposure and behavioral outcomes in children (Appleyard et al., 2005), but cumulative ALE exposure during pregnancy has not been explored. A recent study that included 459 mothers of autistic children showed that maternal exposure to stress during pregnancy may potentially contribute to the degree of the autism‐associated symptoms, however, there was a high risk of recall bias (Alamoudi et al., 2023). Another prospective cohort study found that children were more likely to have more autism‐associated symptoms if their mother had experienced stress, depression, financial or mental health difficulties, or antidepressant use during pregnancy (Seebeck et al., 2023). This study included 3,000 children, of whom 102 (4.3%) scored higher than the threshold for the likelihood of being autistic at age three, based on a screening questionnaire (Seebeck et al., 2023). However, the analysis did not account for familial confounding. In addition, replication is desired in different, larger samples.

Autism and the liability to experience ALEs are both influenced by genetic factors and these genetic factors may overlap, as evidenced by family studies and research utilizing polygenic scores (PGS) (Billig et al., 1996; Havdahl et al., 2021; Plomin et al., 1990). The heritability of autism based on twin studies is estimated as 61%–94% (Tick et al., 2016). Previous studies examining the relationship between ALEs and autism have overlooked unmeasured familial confounding, that is, unobserved or unmeasured genetic or environmental factors that are shared within the family, and which affect both exposure and outcome. This is an important limitation, as previous research using PGSs suggested that mothers with higher polygenic predisposition for neurodevelopmental conditions such as attention‐deficit hyperactivity disorder (ADHD) and autism are more likely to experience ALEs (Havdahl et al., 2021) (Wootton et al., 2017). Consequently, the association between ALEs during the prenatal period – a critical time for brain development (Almond & Currie, 2011; Bateson et al., 2004)‐ and neurodevelopmental conditions in children may be confounded by unmeasured genetic or early‐environmental factors (Carlsson et al., 2021). A recent study utilizing PGS underscored the significance of genetic confounding in studies of hypothesized predisposing factors related to neurodevelopmental conditions (Havdahl et al., 2022). These findings have revealed that some associations previously thought to be causal may actually be influenced by genetic confounding (Havdahl et al., 2022). Utilizing study designs that incorporate data on related individuals, such as sibling comparisons, can help disentangle these causal processes by accounting for unmeasured familial confounding (D’Onofrio et al., 2013).

Using population‐based data from the Norwegian Mother, Father and Child Cohort Study (MoBa) (Magnus et al., 2006, 2016) and the Medical Birth Registry of Norway (MBRN), this study investigated the association between ALEs during pregnancy and the likelihood of autism and autistic‐traits in children. We assessed the association between cumulative ALE exposure and perceived ALE‐related distress during pregnancy and autism and autistic‐like traits in children, using family‐based designs to control for unmeasured familial confounding.

## MATERIALS AND METHODS

### Data sources and study population

MoBa is an ongoing population‐based pregnancy cohort study conducted by the Norwegian Institute of Public Health. Mothers were recruited from all over Norway in 1999–2008 during their first antenatal visit (week 17–20) at the clinic (Brantsæter et al., 2008). The women consented to participation in 41% of the pregnancies. The cohort includes approximately 114,500 children, 95,200 mothers and 75,200 fathers. It is linked with the Medical Birth Registry of Norway (MBRN), which is a national health registry containing information about all births in Norway, and the National Patient register that includes information on all in‐ and outpatient clinic visits. The month of birth was retrieved from Statistics Norway, however, due to data safety the actual date is protected, therefore, we imputed “15” as a day of the month of birth (Kanina et al., 2023). The current study is based on 113,536 children in version 12 of the quality‐assured data files released for research in January 2019. The establishment of MoBa and initial data collection was based on a license from the Norwegian Data Protection Agency and approval from The Regional Committees for Medical and Health Research Ethics. The MoBa cohort is currently regulated by the Norwegian Health Registry Act. The current study was approved by The Regional Committees for Medical and Health Research Ethics (2016/1702).

**FIGURE 1:**
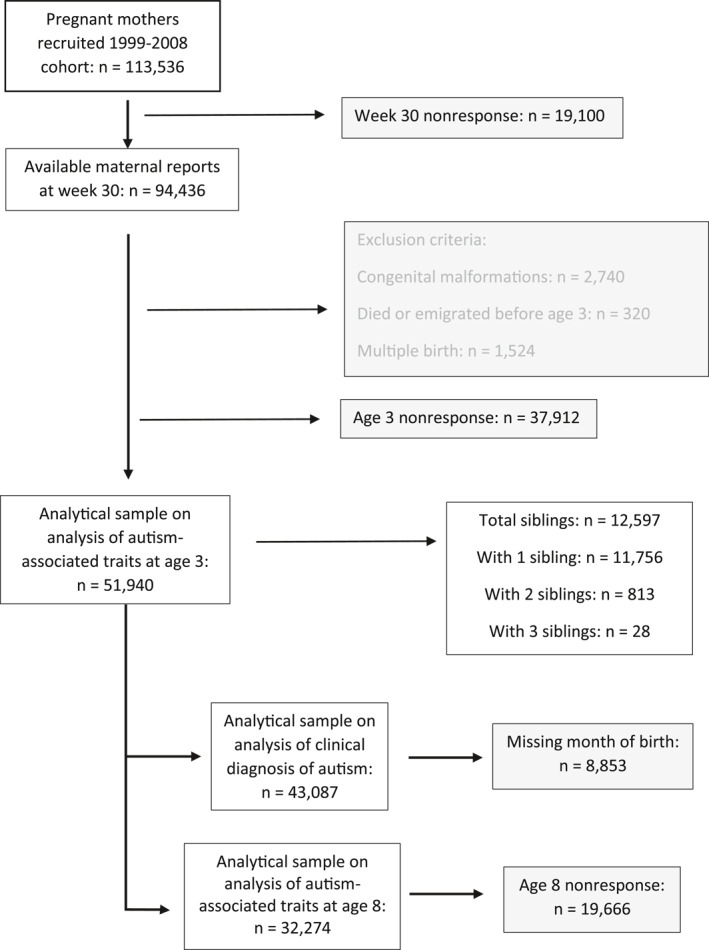
MoBa cohort study population flowchart.

This study includes all singleton children who were born 1999–2009 to mothers who at week 30 during pregnancy completed a questionnaire containing questions on ALEs, and for whom maternal reports on child outcomes at age 3 years were available (Figure 1). The analytical sample includes 56,524 children. Singleton children born to mothers who completed a questionnaire when the children were 8 years old were included in an additional analysis of 32,274 children. We excluded individuals with congenital malformations, stillbirths, or abortions. To investigate the influence of genetic and environmental factors shared by siblings, we also identified a subgroup of siblings (*n* = 12,597), based on their mother’s ID number where mothers participated in MoBa with at least two children.

## MEASURES

### Exposure

Information on the following ALEs experienced during the past 12 months was retrieved from the MoBa questionnaire at week 30 of the pregnancy: problems at work/study place, financial problems, divorce/separation, conflicts or problems with family, friends, or neighbors, being seriously ill or injured, having someone close being seriously ill or injured, being involved in a serious traffic accident, house fire or robbery, experiencing a loss of someone close and other unspecified factors (Coddington, 1972). For each reported ALE, respondents were asked to rate how painful or difficult it was (perception): “not too bad” – one point, “painful/difficult” – two points, “very painful/difficult” – three points. Maternal reports of ALEs were analyzed as two cumulative scores, one for the number of experienced ALEs (ranging from 0 to 9) and one which also considered the perception of the event (possible range from 0 to 27).

### Outcome

#### Autism diagnosis

We identified whether individuals had a clinical diagnosis of autism, retrieved from the National Patient register of Norway between 2008 and 2020, based on the international classification of diseases (ICD)‐10 codes for childhood autism (F84.0), atypical autism (F84.1), Asperger syndrome (F84.5), other pervasive developmental disorders (F84.8), and unspecified pervasive developmental disorder (F84.9).

#### Mother‐rated autism‐associated traits

The social communication questionnaire (SCQ) was filled out when children were 3 and 8 years old. The SCQ is considered to be a reliable instrument for measuring autistic traits (Chesnut et al., 2017). A meta‐analysis showed that children younger than 4 years had lower scores compared to older children (area under the curve: 0.765 and 0.922, respectively) (Chesnut et al., 2017). Based on the SCQ, we derived three continuous outcomes at each age: (a) total score autism‐associated traits based on 39 items, (b) domain score for restricted and repetitive behavior (RRB) that consisted of 12 questions, and (c) domain score for social communication (SC) that consisted of 25 questions.

### Covariates

We included maternal and paternal age at birth (categorized as <20, 21–24, 25–29, 30–34, 35+), child’s year of birth (1999–2004 vs. 2005–2009), child’s region of birth (south/east, west, middle, north) as covariates for the main population and additionally parity for the sibling population and adjusted for them in all analyses.

### Statistical analysis

The association between two cumulative exposure measures of ALEs (i.e., total number of ALEs experienced, and total score of perception of ALEs as painful/stressful) and clinical diagnosis of autism in offspring was assessed with crude and adjusted hazard ratios (HRs) and 95% confidence intervals (CI), using separate Cox proportional hazards regression models for each exposure. We used cluster robust standard errors to account for correlated observations among siblings in the full sample. The participants were followed from birth until the end of follow‐up in 2021, death, migration, or diagnosis of autism. The adjusted analysis accounted for maternal age at birth, paternal age at birth, child’s year of birth, and child’s region of birth in multivariable models.

The associations between the two cumulative exposure measures of ALEs and mother‐rated autism‐associated traits (SCQ full; RRB; and SC) at age 3 years and age 8 years were assessed in 12 separate models (2 exposures × 6 outcomes) with crude and adjusted β‐coefficients and 95% CIs, using linear regression with cluster robust standard errors to account for correlated observations among siblings within the full population, as well as potential non‐normal distribution of the SCQ score. The adjusted analysis accounted for maternal and paternal age at birth, child’s year of birth, and child’s region of birth.

In sibling comparison analyses, comparisons were made between differently exposed siblings to investigate the extent to which the relationships observed in standard analyses could be attributed to unmeasured familial confounding. These shared factors among siblings encompass enduring risk factors present in their mother, as well as 50% of the genetic predisposition for autism and autistic traits that originate from the child. Technically, we carried out the sibling comparison analyses by repeating the full population analyses, now adding a family‐specific baseline hazard to the Cox regression, and a family‐specific intercept to the linear regression. These family‐specific terms absorb, and thereby implicitly adjust for, all factors that are constant within families (Sjölander et al., 2022).

In sibling restricted analyses, we excluded all children who do not have siblings participating in MoBa. Consequently, discrepant results between sibling analyses and full population analyses could also reflect effect modification by family size, that is, the effect of ALEs depending on whether a child has siblings or not. To assess this, we repeated the Cox regressions and linear regressions, now without family‐specific baseline hazards and intercepts, but restricting the analyses to those individuals who had siblings. If effect modification by family size would be negligible, we expect these sibling‐restricted analyses to give similar results as the full population analyses.

### Sensitivity analyses

We performed sex‐stratified Cox regressions between ALEs and autism to see whether there is a difference between the male and female populations. Additional linear regression models (for age 3 and 8 scale outcomes) were fitted for each ALE separately (each coded 0–1) to check for potential differential associations of separate events. We also stratified sibling analyses for the age difference between sibling pairs (age gaps: 0–2 years (*n* = 6522) versus 3–5 years (*n* = 6047)) to investigate whether the association was influenced by varying age differences (Kuja‐Halkola et al., 2019). The missing values of the ALEs scale, the SCQ scale and its subscales, region of birth and the father’s age at birth were addressed in sensitivity analyses by using multiple imputation in Stata via the mi command with 25 datasets created as a part of the imputation process. The variables of diagnosis of autism, parity, year of birth, sex, and mother’s age had no missing data and, therefore, were not imputed but rather included as recorded in the register. Region of birth as a categorical variable was imputed using multinomial logistic regression, the ALEs scale with truncated regression, and all SCQ scales and father’s age with predictive mean matching (Rubin, 2004). We also conducted Spearman's rank correlation to assess the association between the number of ALEs and the proportion of ALEs that were perceived as very painful.

## RESULTS

The study population included 51,940 individuals. 827 (1.6% of the analytical sample) had been diagnosed with autism, of whom 225 were females (27.2%). In the sibling subsample, we identified 11,756 individuals with one sibling, 813 with two and 28 with three siblings. 294 (1.3%) non‐exposed individuals were diagnosed with autism, compared to 533 (1.9%) exposed. Descriptive statistics of the study population are presented in Table 1. The distribution of ALEs between individuals with and without autism diagnosis in the full population was as follows: 0 ALEs (294 vs. 23,149), 1 ALE (245 vs. 15,198), 2 ALEs (167 vs. 8233). In the sibling restricted analysis: 0 ALEs (63 vs. 6101), 1 ALE (61 vs. 3669), 2 ALEs (22 vs. 1836) (See full description in Table S1). The occurrence and perception of the ALEs were not strongly correlated: the absolute numbers of reported ALEs varied from 427 (0.82%) for serious accident to 12,192 (23.47%) for problems at work, and the reports on the severity of the exposure varied as well (see Table S2). There was a moderate positive monotonic relationship between the number of ALEs and the proportion of ALEs that were perceived as “very painful” (*ρ* = 0.2524, *p* = 0.000).

**TABLE 1:** Descriptive information of the analytical sample.

		Full population		Sibling restricted sample	
		Maternal exposure to any adverse life event			
		Non‐exposed	Exposed	Non‐exposed	Exposed
		*N* = 23,443	*N* = 28,497	*N* = 6164	*N* = 6433
Sex	Male	11,927 (45.0%)	14,554 (55.0%)	3202 (49.0%)	3338 (51.0%)
	Female	11,516 (45.2%)	13,943 (54.8%)	2962 (48.9%)	3095 (51.1%)
Birth year	1999–2004	8538 (43.8%)	10,963 (56.2%)	1967 (45.2%)	2389 (54.8%)
	2005–2008	14,905 (45.9%)	17,534 (54%)	4197 (50.9%)	4044 (49.1%)
Clinical diagnosis of autism	Yes	294 (35.6%)	533 (64.4%)	63 (40.1%)	94 (59.9%)
	No	23,149 (45.3%)	27,964 (54.7%)	6101 (49.0%)	6339 (51.0%)
Mother’s age at birth	<20	85 (28.7%)	211 (71.3%)	<20 (41.2%)	20 (58.8%)
	20–24	1660 (37.5%)	2769 (62.5%)	354 (38.4%)	568 (61.6%)
	25–29	7732 (44.7%)	9561 (55.3%)	2161 (48.4%)	2301 (51.6%)
	30–34	9749 (47.1%)	10,947 (52.9%)	2752 (51.0%)	2642 (49.0%)
	35+	4217 (45.7%)	5009 (54.3%)	883 (49.5%)	902 (50.5%)
Father’s age at birth	<20	24 (26.7%)	66 (73.3%)	<20 (50.0%)	<20 (50.0%)
	20–24	647 (34.5%)	1229 (65.5%)	133 (36.6%)	230 (63.4%)
	25–29	5110 (44.0%)	6497 (56.0%)	1347 (46.5%)	1548 (53.5%)
	30–34	9480 (46.0%)	11,134 (54.0%)	2675 (50.0%)	2674 (50.0%)
	35+	8151 (46.2%)	9492 (53.8%)	1997 (50.3%)	1972 (49.7%)
	Missing	31 (28.2%)	79 (71.8%)	<20 (63.6%)	<20 (36.4%)
Region of birth	South‐east	12,062 (44.4%)	15,097 (55.6%)	2749 (47.2%)	3077 (52.8%)
	West	6465 (47,0%)	7290 (53.0%)	2134 (51.5%)	2007 (48.5%)
	Middle	3579 (45.6%)	4271 (54.4%)	965 (49.4%)	987 (50.6%)
	North	1335 (42.1%)	1838 (57.9%)	316 (46.6%)	362 (53.4%)
	Missing	<5	<5		
Parity	0	10,854 (44.1%)	13,741 (55.9%)	2151 (45.4%)	2582 (54.6%)
	1	8272 (46.2%)	9648 (53.8%)	2942 (51.3%)	2793 (48.7%)
	2	3437 (46.3%)	3987 (53.7%)	853 (50.4%)	838 (49.6%)
	3	693 (44.9%)	852 (55.1%)	170 (51.8%)	158 (48.2%)
	4 or more	187 (41.0%)	269 (59.0%)	48 (43.6%)	62 (56.4%)
Age 3 SCQ, mean (SD)		6.0 (3.3)	6.4 (3.4)	5.4 (3.1)	5.8 (3.2)
Age 8 SCQ, mean (SD)		3.3 (2.9)	3.4 (2.9)	3.0 (2.7)	3.0 (2.7)

### Full population analysis

**FIGURE 2:**
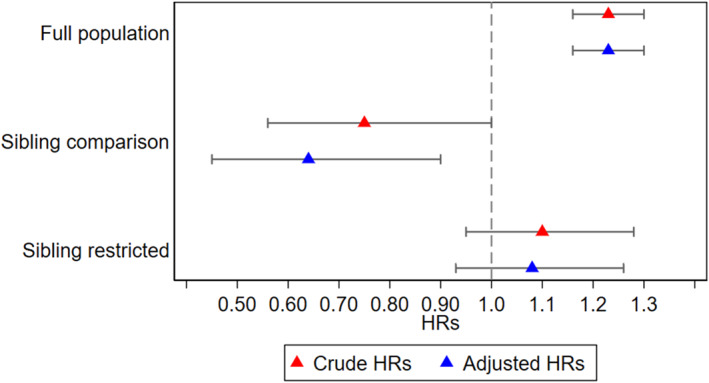
Crude and adjusted hazard ratios for cumulative exposure to ALEs and clinical diagnosis of autism.

**FIGURE 3:**
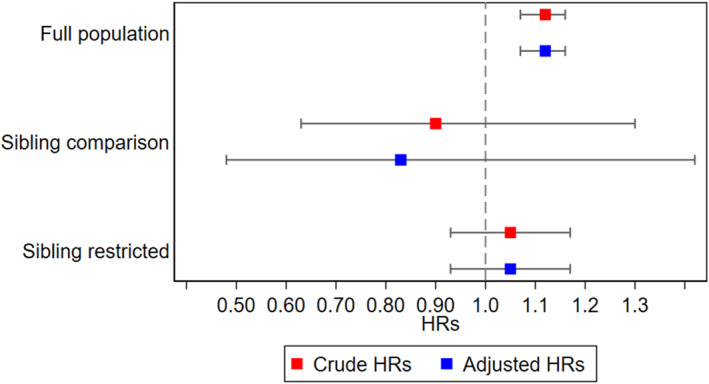
Crude and adjusted hazard ratios for mother’s perception of cumulative exposure to ALE and clinical diagnosis of autism.

In the full sample, children had a 23% increased likelihood of autism diagnosis (HR: 1.23, 95% CI [1.16–1.30]) per each prenatally experienced ALE in the cumulative scale (Figure 2). Adjusting for measured potential confounders (maternal and paternal age at birth, child’s year of birth, child’s region of birth) made little difference (HR: 1.23, 95% CI [1.16–1.30]). Mothers' perception of the ALEs was associated with a 12% increase in likelihood of diagnosed autism (HR: 1.12; 95% CI [1.07–1.16]) per each unit in the cumulative exposure scale of perceived stressfulness in the full sample, when adjusting for parental age at birth, birthyear category and region of birth (Figure 3). Furthermore, a one unit increase in the exposure to cumulative ALEs during pregnancy was associated with a mean increase of 0.24 (95% CI [0.21–0.27]) in the full SCQ score at age 3 years; 0.20 (95% CI [0.18–0.22]) in the RRB score; and 0.03 (95% CI [0.01–0.04]) in the SCD score, in adjusted models (Table 2). At age 8 years, a one unit increase in the cumulative ALEs exposure during pregnancy was associated with a mean increase of 0.07 (95% CI [0.04–0.10]) in the full SCQ score; 0.10 (95% CI [0.09–0.12]) in the RRB; and −0.03 (95% CI [−0.06 to −0.01]) in the SCD score (Table 2). Mother’s perception of the ALEs was not associated with increased SCQ scores at age 3 or 8 years (Table 3).

**TABLE 2:** Crude and adjusted β coefficients for cumulative exposure to adverse life events at week 30 of pregnancy and mother‐rated autism‐associated traits at age 3 and 8.

	Full population analysis		Sibling comparison analysis		Sibling restricted analysis	
	Crude β (95% CI)	Adj β (95% CI)	Crude β (95% CI)	Adj β (95% CI)	Crude β (95% CI)	Adj β (95% CI)
SCQ score, age 3						
Full scale	0.28 (0.25–0.31)	0.24 (0.21–0.27)	0.13 (0.06–0.21)	−0.02 (−0.09–0.05)	0.26 (0.20–0.32)	0.21 (0.15–0.27)
RRB scale	0.23 (0.21–0.25)	0.20 (0.18–0.22)	0.11 (0.06–0.16)	−0.01 (−0.06–0.04)	0.23 (0.18–0.27)	0.18 (0.14–0.23)
SC scale	0.04 (0.02–0.05)	0.03 (0.01–0.04)	0.02 (−0.02–0.06)	−0.01 (−0.05–0.04)	0.03 (−0.00–0.06)	0.01 (−0.02–0.04)
SCQ score, age 8						
Full scale	0.08 (0.05–0.11)	0.07 (0.04–0.10)	0.02 (−0.06–0.10)	0.02 (−0.06–0.10)	0.05 (−0.01–0.12)	0.05 (−0.02–0.11)
RRB scale	0.11 (0.09–0.12)	0.10 (0.09–0.12)	−0.02 (−0.06–0.01)	−0.03 (−0.06–0.01)	0.09 (0.06–0.11)	0.08 (0.05–0.11)
SC Scale	−0.03 (−0.06–0.00)	−0.03 (−0.06–0.01)	0.04 (−0.03–0.11)	0.04 (−0.03–0.11)	−0.03 (−0.09–0.02)	−0.04 (−0.09–0.02)

*Note*: Adjusted for parental age at birth, birthyear category and region of birth.

Abbreviations: CI – confidence interval, RRB – restrictive and repetitive behavior, SC – social communication, SCQ – social communication questionnaire.

**TABLE 3:** Crude and adjusted mean difference for mother’s perception of cumulative exposure to adverse life events at week 30 of pregnancy and Social Communication questionnaire (SCQ) scores at age of 3 and 8 in full population.

	Crude B (95% CI)	Adj B (95% CI)
SCQ score at age 3		
Full scale	0.09 (0.03–0.15)	0.01 (−0.05–0.06)
RRB scale	0.05 (0.00–0.10)	−0.02 (−0.06–0.02)
SC scale	0.04 (0.01–0.07)	0.02 (−0.01–0.06)
SCQ score at age 8		
Full scale	0.02 (−0.04–0.09)	0.02 (−0.04–0.08)
RRB scale	−0.00 (−0.03–0.03)	−0.01 (−0.03–0.02)
SC Scale	0.03 (−0.02–0.08)	0.03 (−0.02–0.08)

*Note*: Adjusted for parental age at birth, birthyear category, region of birth.

Abbreviations: CI – confidence interval, RRB – restrictive and repetitive behavior, SC – social communication, SCQ – social communication questionnaire.

When assessing the effect of ALEs separately, point estimates suggested that financial problems and divorce or separation during pregnancy were associated with a higher likelihood of clinically diagnosed autism, and with higher scores on the full SQC, at both age 3 and 8 years. However, confidence intervals for life events often overlapped (Supporting Tables S3 and S4).

### Sibling comparison analysis

In sibling comparison analyses, prenatal exposure to cumulative ALEs was associated with a decreased likelihood of autism diagnosis (HR: 0.64; 95% CI [0.42–0.98]). In the fully adjusted model, the estimate decreased further (HR: 0.53 (95% CI [0.31–0.90]) (Figure 2). The analysis of the mother’s perception of ALEs during pregnancy in the sibling comparison showed HRs of being diagnosed with autism were 0.90 (95% CI [0.63–1.16]) per each ALE in the cumulative exposure scale and did not change after adjustment for covariates, HR = 0.83 (95% CI [0.48–1.42]) (Figure 3). In sibling models, associations between prenatal exposure to ALEs and mother‐rated autism‐associated traits at age 3 and 8 in siblings were also attenuated toward the null and not statistically significant in adjusted models (Table 2).

### Sibling restricted analysis

In sibling restricted analyses, prenatal exposure to cumulative ALEs was associated with an increased likelihood of autism diagnosis (HR: 1.10; 95% CI [0.95–1.28]) per each ALE in the cumulative exposure scale. In the fully adjusted model, the estimate decreased slightly (HR: 1.08; 95% CI [0.92–1.26]) (Figure 2). The analysis of mother’s perception of ALEs during pregnancy in the subsample of siblings showed HRs of being diagnosed with autism were 1.05 (95% CI [0.93–1.17]) per each step increase on the ALE perception scale according to cumulative exposure scale and did not change after adjustment for covariates 1.05 (95% CI [0.93–1.17]) (Figure 3). Generally, the estimates from the sibling restricted analyses were closer to the full population estimates than the sibling comparison estimates, which indicates that the difference between the latter two was mainly driven by familial confounding.

### Sensitivity analysis

In sex‐stratified analyses, point estimates suggested a slightly higher association between ALE and autism for females than for males, but confidence intervals overlapped (1.27 (1.15–1.39), versus 1.20 (1.13–1.28)) (Table S5). Stratified sibling models by sibling age gap (0–2 vs. more than 3) did not affect the results (Table S6). When analyses were repeated using multiple imputation of missing values, the results did not differ (Table S7).

## DISCUSSION

Based on 51,940 children, we found that a cumulative exposure to ALEs experienced by mothers during pregnancy was associated with an increased likelihood of autism diagnosis and autistic‐like traits in children at the age of 3 and 8 in the full population. This association completely attenuated after adjustment for familial confounding in the sibling comparison. Moreover, similar trends were observed in analyses considering the mother’s perception of cumulative ALEs and clinical diagnosis of autism and autistic‐like traits at the age of 3 and 8, with the association being completely attenuated in sibling comparisons. The results of sibling restricted sample resembled the results in the full population sample, meaning that the subpopulation of siblings was not different from the full population. These results therefore suggest that the association between ALE, autism and autistic traits is at least partly due to genetic and/or shared environmental factors that influence both the likelihood of experiencing ALEs and autism.

Observational studies frequently report associations between various environmental exposures and autism, often suggesting these exposures as “risk” factors (Alamoudi et al., 2023; Seebeck et al., 2023; Tick et al., 2016). However, without accounting for genetic influences, these associations can be misleading (Havdahl et al., 2022; Leppert et al., 2019). A limited body of research has used genetically informative study designs to investigate predisposing factors for neurodevelopmental conditions (Carlsson et al., 2021). A recent systematic review showed that most studies employing such designs, including twin and sibling comparisons, found previously reported associations between early environmental predisposing factors and neurodevelopmental conditions, including autism, to be accounted for by familial confounding (Carlsson et al., 2021). Given the generally heritable nature of autism, it is important to account for unmeasured familial confounding when assessing causality in associations between specific exposures and autism.

There is limited evidence about the associations between ALEs reported by mothers during pregnancy and autism in children. A review of seven articles on prenatal factors found a higher prevalence of cumulative ALEs during pregnancy in individuals with autism; however, the quality of the included articles was low (Dodds, 2021). Previously published results might have been affected by recall bias, and it is likely that the observed associations might be confounded by unmeasured genetic or environmental familial factors. There is a need for further research that addresses these limitations (Alamoudi et al., 2023). Our results, from models not adjusted for familial confounding, align with previous reports linking ALEs and autism in the general population (Li et al., 2023). For example, a study on cumulative stressful life events reported by mothers found increased autistic traits and communication difficulties among children exposed to multiple ALEs during pregnancy (Varcin et al., 2017). Historically, environmental explanations for autism fueled harmful theories and promoted stigmatization of mothers of autistic individuals. Observational studies implying causality between ALEs during pregnancy and autism may perpetuate this stigma. Misinterpretations risk unfairly blaming parents, especially mothers, for their child's diagnosis. Thus, it is crucial to interpret such findings carefully and use research designs that account for confounding. Our study is the first, to our knowledge, to use sibling comparisons to estimate the association between cumulative exposure to ALEs during pregnancy and the mother’s perception of ALE and clinical diagnosis of autism and autistic‐like traits; and our results suggest that previously reported associations may in large part reflect confounding by family‐level factors.

This study has several strengths. We were able to assess both clinical diagnosis of autism, based on linked records from the National Patient Register of Norway, and mother‐rated autistic like traits at two time points. Norwegian pediatric healthcare is publicly funded, allowing universal access to both primary and non‐primary healthcare, including autism diagnostics, mitigating the risk of socially disadvantaged groups remaining underdiagnosed. The rich questionnaire data meant we could also consider the mother’s perception of ALEs. Crucially, we accounted for unmeasured genetic and environmental familial confounding by using sibling comparisons. Sensitivity analyses based on multiple imputed data indicated that missingness in questionnaire data had not influenced conclusions.

Nevertheless, the MoBa cohort represents around 41% of the total Norwegian population, with a higher presence of individuals from higher socio‐economic classes, especially those mothers who have more than one child, which may limit the generalizability of the results (Nilsen et al., 2009). The results of this study should be extrapolated to other settings with caution because Norway has a relatively high level of social security and universal access to healthcare. Due to discrepancies in welfare and healthcare systems compared to those in other countries, the findings in other settings could be different and thus require further research in other contexts. There might be selection bias among the participants due to parity. A Swedish study revealed that families of autistic children did not experience a universal reproductive stoppage after considering birth order and other factors (Kuja‐Halkola et al., 2019). Within‐sample selection bias might have occurred in the sibling subsample due to parent of MoBa participants being more involved in the study (Askelund et al., 2023). Another limitation reflects the time period when ALEs could have happened. The questionnaire administered at week 30 of pregnancy gathers data on ALEs experienced within the 12 months preceding assessment, encompassing a period prior to conception. Nevertheless, the impact of ALEs may persist beyond this window, potentially influencing both early and late stages of pregnancy.

## CONCLUSION

This cohort study sheds light on the association between exposure to cumulative adverse life events in pregnancy, the mother's perception of these events, and autism and autism‐associated traits in children. The results indicate that these associations may not be causal and are confounded by genetic and environmental factors shared within a family. This highlights the importance of using family‐based designs in studies assessing causality in associations between environmental factors and autism diagnosis and autistic‐like traits.

## AUTHOR CONTRIBUTIONS

**Aleksandra Kanina:** Data curation; formal analysis; investigation; methodology; project administration; visualization; writing—original draft; writing—review and editing. **Arvid Sjölander:** Formal analysis; methodology; supervision; writing—review and editing. **Miriam I. Martini:** Investigation; methodology; project administration; writing—original draft; writing—review and editing. **Agnieszka Butwicka:** Conceptualization; methodology; supervision; writing—review and editing. **Henrik Larsson:** Methodology; supervision; writing—review and editing. **Amanda M. Hughes:** Formal analysis; methodology. **Márta K. Radó:** Supervision; writing—review and editing. **Mark J. Taylor:** Methodology; project administration; supervision; writing—review and editing. **Alexandra Havdahl:** Conceptualization; data curation; funding acquisition; methodology; project administration; resources; writing—review and editing. **Helga Ask:** Conceptualization; data curation; formal analysis; methodology; project administration; resources; software; writing—review and editing **Mina A. Rosenqvist:** Conceptualization; data curation; formal analysis; funding acquisition; investigation; methodology; project administration; resources; supervision; writing—original draft; writing—review and editing.

## CONFLICT OF INTEREST STATEMENT

H. L. reports receiving grants from Shire Pharmaceuticals; personal fees from and serving as a speaker for Medice, Shire/Takeda Pharmaceuticals and Evolan Pharma AB; all outside the submitted work. H. L. is editor‐in‐chief of JCPP Advances.

## ETHICAL CONSIDERATIONS

The current study was approved by The Regional Committees for Medical and Health Research Ethics (2016/1702).

## Supporting information

Supplementary Material

## Data Availability

The MoBa data are not publicly available as the consent given by the participants does not permit the for storage of data on an individual level in repositories or journals. Researchers who want access to data sets for replication should submit an application to datatilgang (at)fhi.no. Access to datasets requires approval from The Regional Committee for Medical and Health Research Ethics in Norway and an agreement with MoBa.
