# Supplementary material for: Prenatal exposure to adverse life events and autism and autistic‐like traits in children in the Norwegian Mother, Father and Child Cohort Study (MoBa)
Source: JCPP Adv. 2025 Mar 22;5(4):e70002. doi: 10.1002/jcv2.70002 (PMC12698280; doi:10.1002/jcv2.70002)
Supplement: Supplementary file 1 — Supplementary Material [file JCV2-5-e70002-s001.docx]

**Supporting Information**

**Prenatal exposure to adverse life events and autism and autistic-like traits in children in the Norwegian Mother, Father and Child Cohort Study (MoBa)**

Aleksandra Kanina^1^, Arvid Sjölander^1^, Miriam I. Martini^1^, Agnieszka Butwicka^1,2,3,4^, Henrik Larsson^1,5^, Amanda M Hughes^6^, Márta K. Radó^1^, Mark J. Taylor^1^, Alexandra Havdahl^7,8,9^, Helga Ask^7,8^, Mina A. Rosenqvist^1^

1. Department of Medical Epidemiology & Biostatistics, Karolinska Institutet, Stockholm, Sweden
2. Division of Mental Health Services, Akershus University Hospital, Lørenskog, Norway
3. Institute of Clinical Medicine, University of Oslo, Oslo, Norway
4. Department of Biostatistics and Translational Medicine, Medical University of Lodz, Lodz, Poland
5. School of medical sciences, Örebro University, Örebro, Sweden
6. MRC Integrative Epidemiology Unit, University of Bristol, Bristol, UK
7. PsychGen Center for Genetic Epidemiology and Mental Health, Norwegian institute of Public Health, Oslo, Norway
8. PROMENTA Research Center, Department of Psychology, University of Oslo, Norway
9. PaGE Research Group, Lovisenberg Diaconal Hospital, Oslo, Norway

**Table S1.** Distribution of adverse life events in the population in relation to diagnosis of autism.

|  | Full population | | Sibling restricted population | |
| --- | --- | --- | --- | --- |
| Number of adverse life events at week 30 | Clinical diagnosis of autism | | | |
|  | No | Yes | No | Yes |
| 0 | 23,149 | 294 | 6,101 | 63 |
| 1 | 15,198 | 245 | 3,669 | 61 |
| 2 | 8,233 | 167 | 1,836 | 22 |
| 3 | 3,243 | 87 | 634 | 9 |
| 4 | 1,001 | 25 | 171 | <5 |
| 5 | 130 | 6 | 9 | <5 |
| 6 | 127 | <5 | 17 | <5 |
| 7 | 23 | 0 | <5 | <5 |
| 8 | 7 | 0 | <5 | <5 |
| 9 | <5 | 0 |  |  |

**Table S2**. Distribution of reports on ALEs and it´s perception.

| **Adverse life event** | | **Perception of adverse life event** | | | |
| --- | --- | --- | --- | --- | --- |
|  | **Reported (%)** | **Missing or misreported** | **Not so bad (%)** | **Painful/ difficult (%)** | **Very painful/ difficult (%)** |
| Problems at work/school | 12,192 (23.47%) | 38,570  (74.26%) | 7,817 (15.05%) | 4,303  (8.28%) | 1,250  (2.41%) |
| Financial problems | 7,554 (14.54%) | 43,635  (84%) | 5,971 (11.50%) | 1,799  (3.46%) | 535  (1.03%) |
| Divorce, separation | 820  (1.58%) | 51,117  (98.41%) | 211  (0.41%) | 268  (0.52%) | 344  (0.66%) |
| Conflicts with family, friends, neighbors | 9,536 (18.36%) | 41,998  (80.84%) | 5,050  (9.72%) | 3,689  (7.10%) | 1,203  (2.32%) |
| Serious illness or injury | 1,246 (2.40%) | 50,611  (97.44%) | 512  (0.99%) | 554  (1.07%) | 263  (0.51%) |
| Anyone close seriously ill or injured | 8,531 (16.42%) | 43,791  (84.30%) | 2,051  (3.95%) | 4,038  (7.77%) | 2,060  (3.97%) |
| Serious traffic incident, fire or robbery | 427  (0.82%) | 51,498  (99.15%) | 246  (0.47%) | 134  (0.26%) | 62  (0.12%) |
| Loss of a close person | 5,835 (11.23%) | 46,530  (89.58%) | 1,162  (2.24%) | 2,422  (4.66%) | 1,826  (3.52%) |
| Other | 1,280 (2.46%) | 50,488  (97.20%) | 207  (0.40%) | 763  (1.47%) | 482  (0.93%) |

**Table S3.** Crude and adjusted hazard ratios for exposure to each adverse life event and clinical diagnosis of autism in the full population.

| **Adverse life event** | **Crude HRs** (95% CI) | **Adjusted* HRs** (95% CI) |
| --- | --- | --- |
| Problems at work/school | 1.40 (1.19 – 1.66) | 1.41 (1.19 – 1.67) |
| Financial problems | 1.71 (1.42 – 2.06) | 1.70 (1.40 – 2.05) |
| Divorce/ separation | 2.57 (1.63 – 4.07) | 2.49 (1.58 – 3.95) |
| Conflicts with family, friends or neighbors | 1.34 (1.11 – 1.60) | 1.34 (1.12 – 1.61) |
| Serious illness or injury | 1.28 (0.82 – 2.00) | 1.28 (0.82 – 1.99) |
| Anyone close seriously ill or injured | 1.13 (0.93 – 1.38) | 1.13 (0.93 – 1.38) |
| Serious traffic accident, house fire or robbery | 0.94 (0.39 – 2.27) | 0.93 (0.39 – 2.25) |
| Loss of a close person | 1.06 (0.85 – 1.34) | 1.05 (0.83 – 1.34) |
| Other | 2.33 (1.64 – 3.31) | 2.32 (1.63 – 3.30) |
| *Adjusted for parental age at birth, birthyear category, region of birth.  CI – confidence interval | | |

**Table S4.** Crude and adjusted β coefficients for exposure to each adverse life event and mother-rated full SCQ score at age 3 and 8 in the full population.

| **Adverse life event** | **Age 3** | | **Age 8** | |
| --- | --- | --- | --- | --- |
|  | **Crude β**  **(95% CI)** | **Adjusted β**  **(95% CI)** | **Crude β**  **(95% CI)** | **Adjusted β**  **(95% CI)** |
| Problems at work/school | 0.38  (0.31 – 0.45) | 0.35  (0.29 – 0.42) | 0.01  (-0.06 – 0.09) | 0.02  (-0.06 – 0.09) |
| Financial problems | 0.77  (0.68 – 0.85) | 0.56  (0.47 – 0.64) | 0.27  (0.17 – 0.37) | 0.23  (0.13 – 0.33) |
| Divorce/ separation | 0.95  (0.71 – 1.20) | 0.64  (0.39 – 0.89) | 0.67  (0.36 – 0.97) | 0.57  (0.27 – 0.88) |
| Conflicts with family, friends, or neighbors | 0.41  (0.34 – 0.49) | 0.37  (0.29 – 0.45) | 0.17  (0.09 – 0.26) | 0.16  (0.07 – 0.24) |
| Serious illness or injury | 0.54  (0.35 – 0.74) | 0.50  (0.31 – 0.70) | 0.08  (-0.14 – 0.29) | 0.04  (-0.17 – 0.24) |
| Anyone close is seriously ill or injured | 0.27  (0.19 – 0.35) | 0.27  (0.19 – 0.35) | 0.05  (-0.04 – 0.14) | 0.05  (-0.04 – 0.13) |
| Serious traffic accident, house fire, or robbery | 0.50  (0.15 – 0.84) | 0.38  (0.04 – 0.72) | 0.09  (-0.26 – 0.44) | 0.08  (-0.27 – 0.43) |
| Loss of a close person | 0.27  (0.18 – 0.36) | 0.22  (0.13 – 0.31) | 0.05  (-0.05 – 0.16) | 0.05  (-0.05 – 0.15) |
| Other | 0.31  (0.11 – 0.51) | 0.32  (0.12 – 0.52) | 0.30  (0.07 – 0.52) | 0.25  (0.03 – 0.48) |

**Table S5.** Crude and adjusted hazard ratios for cumulative exposure to adverse life events and clinical diagnosis of autism in males and females separately.

| **Sex** | **Crude HRs (95% CI)** | **Adjusted HRs (95% CI)** |
| --- | --- | --- |
| Males | 1.20 (1.13 – 1.28) | 1.19 (1.12 – 1.27) |
| Females | 1.27 (1.15 – 1.39) | 1.26 (1.15 – 1.39) |
| Adjusted for parental age at birth, birthyear category, parity, region of birth; CI - confidence interval. CI – confidence interval | | |

**Table S6.** Crude and adjusted hazard ratios for cumulative exposure to adverse life events and clinical diagnosis of autism, stratified by age differences between siblings.

| **Age difference between siblings in a family** | **Crude HRs (95% CI)** | **Adjusted HRs (95% CI)** |
| --- | --- | --- |
| 0 – 2 years | 0.70 (0.47 – 1.06) | 0.62 (0.39 – 1.00) |
| More than 3 years | 0.80 (0.53 – 1.21) | 0.60 (0.34 – 1.04) |
| Adjusted for parental age at birth, birthyear category, parity, region of birth.  CI – confidence interval | | |

**Table S7.** Crude and adjusted β coefficients for cumulative exposure to adverse life events at week 30 of pregnancy and Social Communication questionnaire (SCQ) scores at age of 3 and 8 using multiple imputation.

| **SCQ score at age 3** | **Crude β (95% CI)** | **Adjusted β (95% CI)** |
| --- | --- | --- |
| Full scale | 0.28 (0.25 – 0.31) | 0.24 (0.21 – 0.26) |
| RRB scale | 0.23 (0.21 – 0.25) | 0.20 (0.17 – 0.22) |
| SC scale | 0.04 (0.02 – 0.06) | 0.03 (0.01 – 0.05) |
| **SCQ score at age 8** |  |  |
| Full scale | 0.10 (0.07 – 0.13) | 0.09 (0.06 – 0.12) |
| RRB scale | 0.11 (0.10 – 0.12) | 0.10 (0.09 – 0.11) |
| SC scale | -0.01 (-0.03 - 0.02) | -0.02 (-0.04 - -0.01) |
| Adjusted for parental age at birth, birthyear category, region of birth.  SCQ – social communication questionnaire, RRB - restrictive and repetitive behavior, SC - social communication, CI – confidence interval | | |
